# An Account of Diseases in an Eastern District of London, from Feb. 20, to March 20, 1811

**Published:** 1811-04

**Authors:** 


### *An Account of Diseases in an Eastern District of London, from Feb. 20, to March 20, 1811.*

| | |
|---|---|
| **ACUTE DISEASES.** | |
| Perepneumonia Notha | 5 |
| Pleuritis - | 2 |
| Ephemera - | 1 |
| Cynanche tonsillaris | 2 |
| Rheumatismus acutus | 7 |
| **CHRONIC DISEASES.** | |
| Tussis - | 20 |
| Dyspnœa - | 12 |
| Tussis cum Dyspnœa | 15 |
| Phthisis pulmonalis | 5 |
| Amenorrhœa - | 7 |
| Menorrhagia - | 5 |
| Leucorrhœa - | 10 |
| Enterodynia - - | 4 |
| Obstipatio - | 3 |
| Dysenteria - | 4 |
| Hydrops pectoris - | 3 |
| Hæmorrhois - | 2 |
| Hemiplegia - | 1 |
| Scrophula - | 4 |
| Rheumatismus acutus | 24 |
| **PUERPERAL DISEASES.** | |
| Menorrhagia lochealis | 5 |
| Dolores post Partum | 7 |
| Dysuria - | 3 |
| Enterodynia - | 4 |
| **INFANTILE DISEASES.** | |
| Convulsio - - | 3 |
| Erysipelas infantile | 4 |
| Tabes Mesenterica - | 5 |
| Aphthæ - - | 4 |

The great similarity in the general state of the diseases which have lately occurred, leaves little or no room for remarks on the subject. Diseases of the chest and rheumatism still engage a considerable siderable portion of medical attention, but amongst these no circumstances have arisen, nor have any particular symptoms occurred worthy of any particular remark or attention.

